# Alanine substitution in cellobiohydrolase provides new insights into substrate threading

**DOI:** 10.1038/s41598-017-16434-x

**Published:** 2017-11-24

**Authors:** Shigenobu Mitsuzawa, Maiko Fukuura, Satoru Shinkawa, Keiichi Kimura, Tadaomi Furuta

**Affiliations:** 1 Fundamental Technology Research Center, Honda R&D Co., Ltd., 1-4-1 Chuo, Wako-shi, Saitama 351-0113 Japan; 2 School of Life Science and Technology, Tokyo Institute of Technology, B-62 4259 Nagatsuta-cho, Midori-ku, Yokohama 226-8501 Japan; 3 Present Address: Honda Research Institute Japan Co., Ltd., 8-1 Honcho, Wako-shi, Saitama 351-0188 Japan

## Abstract

The glycoside hydrolase family 7 (GH7) member cellobiohydrolase (CBH) is a key enzyme that degrades crystalline cellulose, an important structural component of plant cell walls. As GH7 CBH is a major component in the enzyme mixture used to degrade biomass into fermentable glucose in biorefineries, enhancing its catalytic activity will significantly impact development in this field. GH7 CBH possesses a catalytic tunnel through which cellulose substrates are threaded and hydrolysed. Despite numerous studies dissecting this processive mechanism, the role of amino acid residues in the tunnel remains not fully understood. Herein, we examined the respective contributions of nine amino acid residues in the catalytic tunnel of GH7 CBH from *Talaromyces cellulolyticus* by substitution with alanine. As a result, N62A and K203A mutants were found to possess significantly higher cellulase activities than wild type. Molecular dynamics simulations showed that the N62 residue interacted strongly with the cellulose substrate, impeding threading, while the N62A mutant allowed cellulose to proceed more smoothly. Furthermore, the W63 residue was observed to facilitate twisting of the cellulose substrate in our simulations. This study helps elucidate cellulose threading and provides insight into biomass hydrolysis.

## Introduction

Plant cell wall is a promising feedstock for next-generation biofuels^1–3^. Its use could diminish the food versus fuel debate associated with conventional biofuels produced from edible biomasses, such as corn starch^4^. Cellulose, the main component in plant cell walls, is a linear molecule composed of glucose units. Cellulases can decompose cellulose into fermentable individual glucose units through hydrolysis. In nature, mixtures that comprise several tens of different enzymes secreted by some fungi and bacteria synergistically are used to hydrolyse recalcitrant crystalline cellulose. In contrast, conventional starch-based biofuel production uses only a few enzymes for biomass hydrolysis^5^. The recalcitrance of cellulose is a critical obstacle to the commercialisation of cellulosic biofuel. In a cost estimation of ethanol production from corn stover, cellulase in substrate hydrolysis contributed $0.34/gal of ethanol product to the total cost of $2.15/gal^6^, which is almost eight times higher than the cost of amylase used in ethanol production from shelled corn^7^. Therefore, enhanced cellulase activity, which would lead to reduced enzyme costs, is greatly needed.

As enzyme cost is the product of the amount used and the production cost per unit amount, the enzyme cost can be reduced by separately lowering these two factors. Previously, we constructed synthetic mixtures consisting of three cellulase components from *Talaromyces cellulolyticus* and two additive hemicellulases from different organisms^8,9^. *Talaromyces cellulolyticus* is a cellulose-degrading fungus originally isolated in Japan^10–12^ and regarded as a promising major source of commercial cellulases^13^. This defined enzymatic cocktail gave a higher sugar yield than that of the culture supernatant of *T. cellulolyticus* at the same dosage. Therefore, a targeted sugar yield can be obtained with a lower amount of enzyme using the synthetic cocktail than the native mixture. To reduce production costs, we constructed *Aspergillus oryzae* strains that were genetically modified to overproduce these enzyme components^14^. To further decrease the amount of enzyme used for saccharification, we aimed to improve the components in the cocktail. In the synthetic cocktail, the glycoside hydrolase family 7 (GH7) member cellobiohydrolase (CBH) made up 47% of the total enzyme content in the optimised composition^15^. GH7 CBH is also the most abundant component in typical cellulolytic enzyme systems expressed by fungi^16^. Therefore, we resolved to improve this enzyme using molecular engineering before focusing on other components. The amount of enzyme required for saccharification is affected by certain properties of the enzyme species employed, including specific activity, degree of product inhibition, and thermal stability. Of these properties, specific activity is the most dominant. Therefore, in this study, we have attempted to enhance the specific activity of GH7 CBH from *T. cellulolyticus* (*Tc*Cel7A).

GH7 CBH from *Phanerochaete chrysosporium* (*Pc*Cel7C) has previously been genetically modified^17^. *Pc*Cel7C variants with single site-directed mutations were constructed on and near the catalytic tunnel using an *in vitro* expression system, and demonstrated that some mutants possessed higher activities than those of wild type (WT) toward phosphoric acid swollen cellulose (PASC) and 4-methylumbelliferyl β-D-cellobioside (MUC)^18,19^. However, the molecular mechanism for the effect of amino acid mutations on enzymatic activity has yet to be elucidated. The molecular structure and catalytic mechanism of GH7 CBH from *Trichoderma reesei* (*Tr*Cel7A) have been studied most intensively^20,21^. GH7 CBH cleaves cellobiose at the reducing end of cellulose substrates sequentially as it slides along the cellulose surface through a processive mechanism^22,23^. Upon enzyme attachment to the surface of crystalline cellulose, the polysaccharide chain initiates threading into the catalytic tunnel. The enzyme then successively repeats the cycle of hydrolysis, product expulsion, and threading^24^. Therefore, threading is a key process in the processive mechanism determining the efficiency and reaction rate of overall degradation^25,26^. The driving force behind this process has been a topic of intense investigation. Based on the crystal structure of *Tr*Cel7A, the catalytic tunnel is 50 Å long and contains nine glucosyl binding subsites (−7 to + 2), with catalytic sites located around subsites −1 and + 1^27^. The interaction between the substrate and the long catalytic tunnel is considered to govern both stepwise movement and hydrolytic activity^28,29^. The strongest interaction has been suggested to occur at the expulsion site (subsites + 1 and + 2)^30,31^, while the next most important location is the first half of the tunnel (subsites −7 to −3)^29^. Subsites −7 to −3 are aligned by hydrogen bond-forming amino acid residues and tryptophan residues, providing hydrophobic stacking interactions. Those residues are thought to guide the cellulose strand toward the catalytic site. To achieve a smooth processive movement, these interactions should be fine-tuned. The importance of the tryptophan residue (W40) at the entrance of the tunnel (subsite −7) of *Tr*Cel7A in guiding cellulose strands into the catalytic tunnel has been confirmed by replacing the residue with alanine^32^. Furthermore, the tryptophan residue (W38) near subsite −4 has been replaced with alanine to elucidate the rate-limiting step of *Tr*Cel7A^33^. Aromatic residues that line the catalytic tunnels of chitinase, another processive glycoside hydrolase, have also been shown to affect processivity by mutating their residues to alanines^34,35^. However, a comprehensive understanding of interactions between glucosyl units situated at subsites and amino acid residues along the tunnel has yet to be obtained. In contrast to tryptophan residues, hydrogen-bond-forming residues have received relatively little attention regarding their effect on threading. Although molecular simulations on *Tr*Cel7A have demonstrated that electrostatic interactions with conserved polar residues at subsites + 2 and −1 provide the thermodynamic driving force behind cellulose chain translocation^36^, other polar residues on the catalytic tunnel have not been investigated. Kostylev *et al*.^37^ reported no significant change in catalytic activity when asparagine and threonine residues at the tunnel entrance of a processive GH48 exocellulase from *Thermobifida fusca* (*Tf*Cel48A) were mutated to alanines.

In this study, we conducted alanine scanning experiments on *Tc*Cel7A^38^ for nine amino acids located along subsites −7 to −3 with the aims of obtaining mutants with higher specific activities and understanding the role of these residues in the enzyme catalytic mechanism. Wild type and mutant *Tc*Cel7A were subjected to activity assays with commercially available microcrystalline cellulose and pretreated corn stover. Furthermore, we performed molecular dynamics simulations for WT and a selected alanine-mutant of *Tc*Cel7A to further elucidate the molecular mechanism.

## Results

### Structure of *Tc*Cel7A

**Figure 1:**
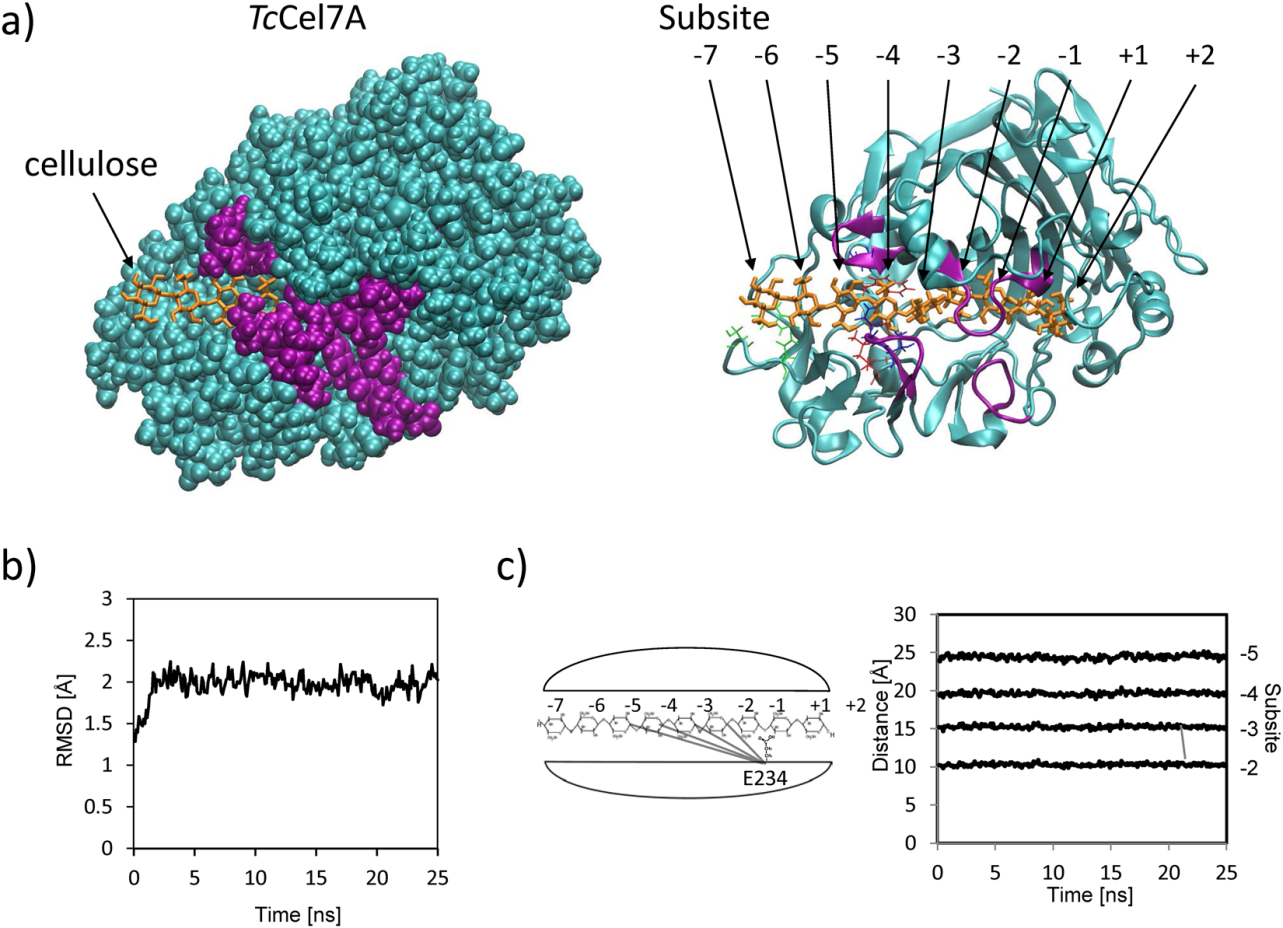
Model structure of *Tc*Cel7A containing cellulose in the catalytic tunnel and its MD simulation. (**a**) Overviews in VDW presentation (left) and in ribbon presentation (right). Four loop regions surrounding the substrate cellulose chain are depicted in purple. (**b**) Time evolution of RMSD from the initial structure and (**c**) of distances between each subunit and the catalytic residue (E234).

First, we constructed a homology model for *Tc*Cel7A using *Tr*Cel7A (PDB:8CEL^27^) as the template (Fig. 1a, Supplementary Movie 1) because the structure of this enzyme has yet to be solved. The amino acid sequence of *Tc*Cel7A showed high similarity with that of *Tr*Cel7A (64% identity and 90% similarity) (Supplementary Fig. S1) and the resultant model structure of *Tc*Cel7A was very similar to the structure of *Tr*Cel7A, with a root mean square deviation (RMSD) between main chain atoms of 1.06 Å (Supplementary Fig. S2). We next conducted a preliminary 25-ns MD simulation of *Tc*Cel7A in water. The resultant RMSD from the initial structure was almost constant, at around 2 Å, throughout the time evolution, indicating that the conformation of the enzyme–substrate complex was stable (Fig. 1b). Furthermore, the distances between each subunit and the catalytic residue (E234) were almost constant (Fig. 1c).

*Tc*Cel7A possessed a tunnel-like structure with four loop regions surrounding the substrate cellulose chain, which is typical of GH7 CBH^26^, although the upper left loop, shown in Fig. 1a, was three amino acids shorter than that in *Tr*Cel7A (Supplementary Figs S1 and S2, Loop 1). In the model structure (Fig. 1a), the cellulose chain had an extended conformation at subsites −7 to −4 that was relatively close to that of crystalline cellulose. However, the cellulose chain was then radically twisted at subsites −3 and −2 prior to the catalytic site (−1/ + 1). This feature suggested that amino acid residues along the tunnel interacted with and applied torsional force to the substrate chain, propelling it further into the catalytic tunnel. This characteristic conformation of the cellulose chain was also confirmed during the above MD simulation (Supplementary Fig. S3).

**Figure 2:**
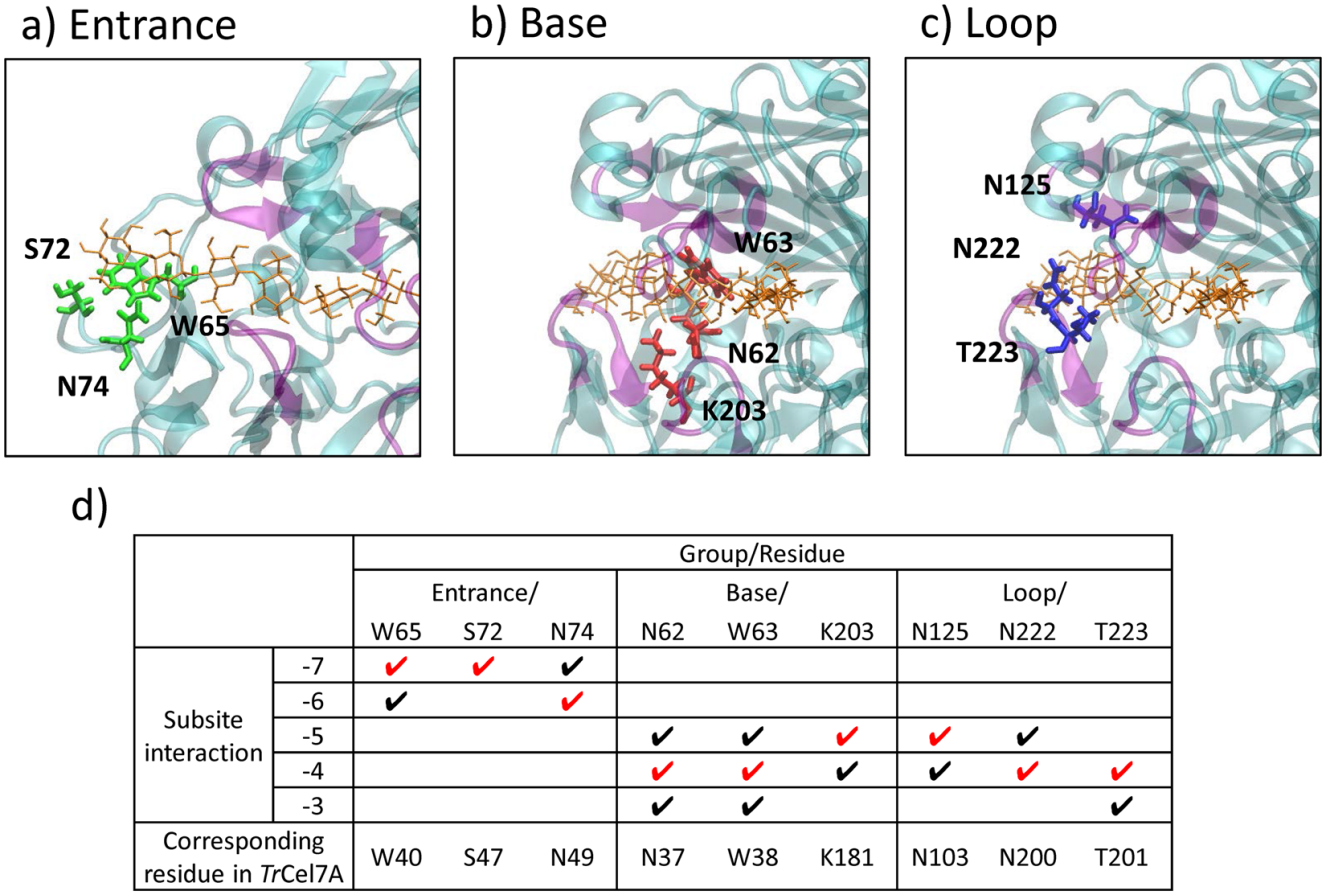
Nine amino acid residues used in the alanine scanning experiment at three locations: (**a**) Entrance, (**b**) Base, and (**c**) Loop. (**d**) Interactions of the nine alanine-scanning residues with subsites. Interactions are indicated by check marks, with the main interactions shown in red for each residue. The corresponding residues in *Tr*Cel7A are listed.

Among these structural features, we concentrated on subsites −7 to −3 in this study, examining interactions between amino acid residues and the substrate. Therefore, we selected nine amino acid residues in this region for alanine scanning experiments. These amino acids were close (within 4 Å) to subsites −7 to −3 and were classified into three groups according to their location in the molecular structure: Entrance (Fig. 2a), Base (Fig. 2b), and Loop (Fig. 2c). Interactions of the nine alanine residues with subsites are summarised in Fig. 2d.

### Production of *Tc*Cel7A proteins

**Figure 3:**
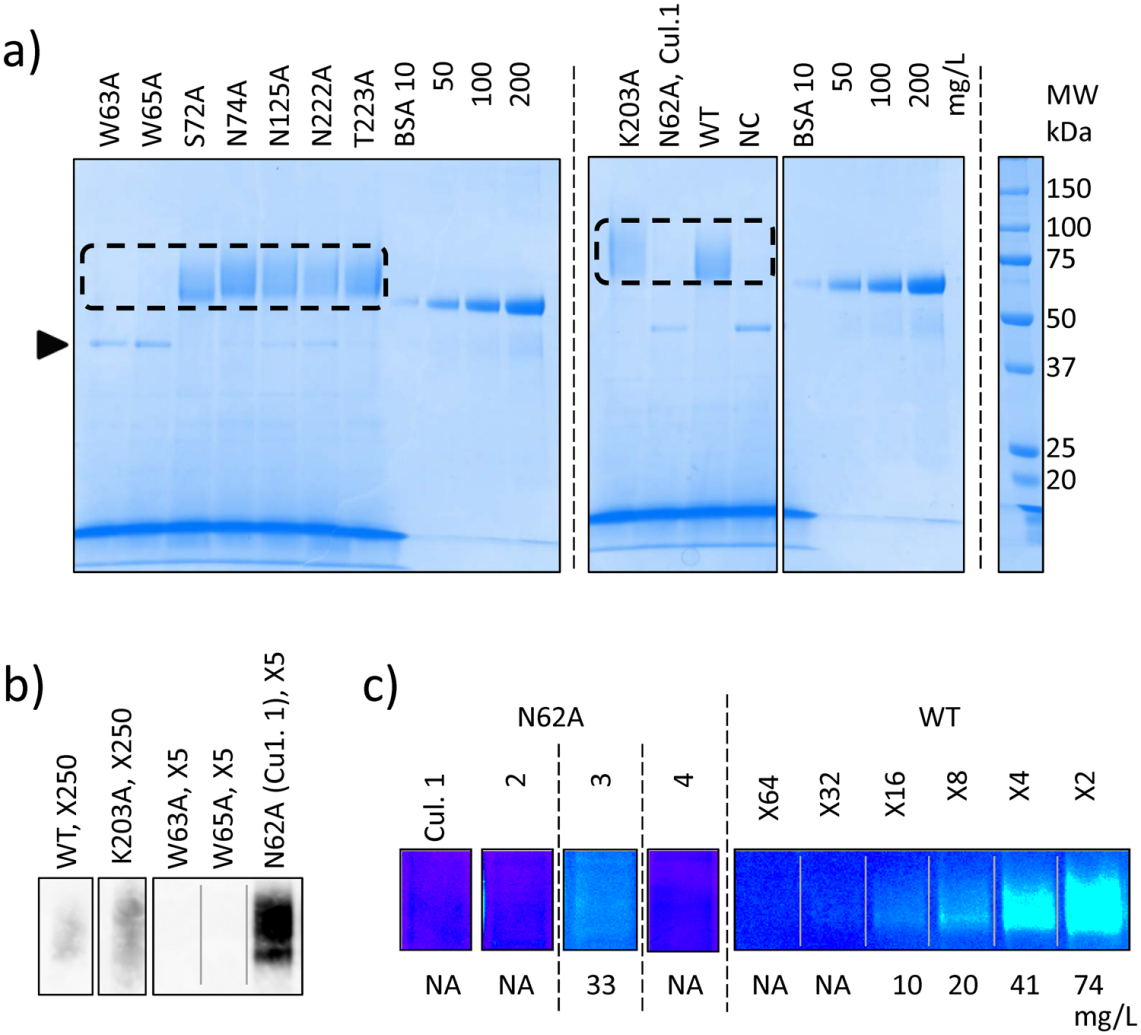
Production of wild-type (WT) and nine alanine-substituted mutants of *Tc*Cel7A. (**a**) SDS-PAGE of WT and nine mutants. NC: untransformed *A. oryzae* strain NS4DLP. BSA concentrations: 10, 50, 100, and 200 mg/L. Target CBHs are shown in a dashed square and the α-amylase position is denoted with a wedge. Full-length gels are presented in Supplementary Fig. S2. (**b**) Western blotting of WT, K203A, W63A, W65A, and N62A. Dilution ratios follow the name for each sample. All images are excerpts from an identical membrane (Supplementary Fig. S3). (**c**) Pseudo-coloured SDS-PAGE images of N62A from four different cultures and of WT with serial dilutions (×2–64) for comparison. For each lane, concentration was determined using image densitometry. Full-length gels with original colour are presented in Supplementary Fig. S4.

Prior to this study, we first attempted to express *Tc*Cel7A WT in four microorganism species, namely *Escherichia coli*, *Bacillus brevis*, *Pichia pastoris*, and *A. oryzae*, with only *A. oryzae* able to produce the protein. Therefore, in this study, WT and nine alanine-substituted mutants of *Tc*Cel7A were produced by employing *A. oryzae* strain NS4DLP as an expression host (Fig. 3). The production of each target protein was assayed using sodium dodecyl sulfate polyacrylamide gel electrophoresis (SDS-PAGE) of the culture supernatant (Fig. 3a, Supplementary Fig. S4). WT and six mutants (S72A, N74A, N125A, N222A, T223A, and K203A) were clearly recognisable in relative abundance. In contrast, W63A, W65A, and N62A mutants were not detected as visible bands. When treated with PNGase F, *Tc*Cel7A WT showed a narrower band on SDS-PAGE (Supplementary Fig. S5), suggesting that the recombinantly produced protein was *N*-glycosylated by the *A. oryzae* host^39^. It was confirmed that *N*-glycosylation did not affect the weight specific activity in comparison of the WT’s, purified form the culture supernatant of *T. cellulolyticus* and recombinantly produced by *A. oryzae*. To examine the production of W63A, W65A, and N62A mutants, we conducted western blotting (Fig. 3b, Supplementary Fig. S6). WT and K203A were also assayed as positive controls. Based on the gel, we confirmed N62A production, but not that of W63A or W65A.

In a preliminary trial to treat mutant proteins including K203A and N62A with ultrafiltration (centrifugal concentration/purification), loss of activity was found that was not observed with WT. In this study, with the aim of obtaining new insight into substrate threading rather than rigorously characterising the highly active mutants, we characterised the mutant proteins only as the culture supernatant of production host strain without any concentration or purification treatments. As the culture supernatant samples contained several proteins other than *Tc*Cel7As and the fragmental amino acids/peptides, as shown Fig. 3a, quantification of the target proteins was carried out using SDS-PAGE image data. In comparison with the serial dilution of WT on SDS-PAGE gel, we determined the concentrations of four cultures of N62A (Fig. 3c, Supplementary Fig. S7). The yields of cultures 1, 2, and 4 were below 5 mg/L. For culture 3, N62A was discernible on the SDS-PAGE gel, allowing the concentration to be determined (33 mg/L, Supplementary Fig. S8). Table 1 summarises the secreted amounts of N62A (Culture 1) and other target proteins.

**Table 1:** Production and hydrolytic activity of WT and nine alanine-substituted mutants of *Tc*Cel7A.

	NC	WT	Entrance/			Base/			Loop/		
			W65A	S72A	N74A	N62A (Culture 1)	W63A	K203A	N125A	N222A	T223A
Production [mg/L]	<5	149	<5	171	229	<5	<5	117	168	126	213
Cellobiose	ND	0.778 (0.01)	ND	0.759 (0.016)	0.860 (0.026)	0.100 (0.013)	ND	0.768 (0.033)	0.882 (0.037)	0.603 (0.032)	0.703 (0.044)
Activity	ND	1.00 (0.01)	ND	0.851 (0.02)	0.720 (0.02)	>**3.85 (0.49)**	ND	**1.26 (0.05)**	1.01 (0.04)	0.908 (0.05)	0.634 (0.04)

Production refers to the concentration (mg/L) of each enzyme obtained from the SDS-PAGE image in Fig. 3a. Cellobiose refers to the HPLC peak area of cellobiose (arbitrary unit) delivered by *Tc*Cel7A in the activity assay. Activity refers to hydrolytic activity, derived from dividing the Cellobiose value by the Production value. Average Cellobiose and Activity values are listed with standard deviations in parentheses. ND: Not detected. Higher activities are shown in bold (N62A and K203A).

### Hydrolytic activity

From secretome analysis by SDS-PAGE (Fig. 3a), we found that, other than CBH, the culture supernatant contained a single detectable band identified as α-amylase of the *A. oryzae* host from its molecular weight^40^. Using the culture supernatant of the untransformed strain (NC), we confirmed that this protein exhibited no cellulase activity on Avicel, carboxymethylcellulose (CMC), or 4-nitrophenyl β-D-glucopyranoside (pNPG). Therefore, we conducted the following experiments employing these culture supernatants (0.22-μm filtrated, but unpurified) as CBH samples.

**Figure 4:**
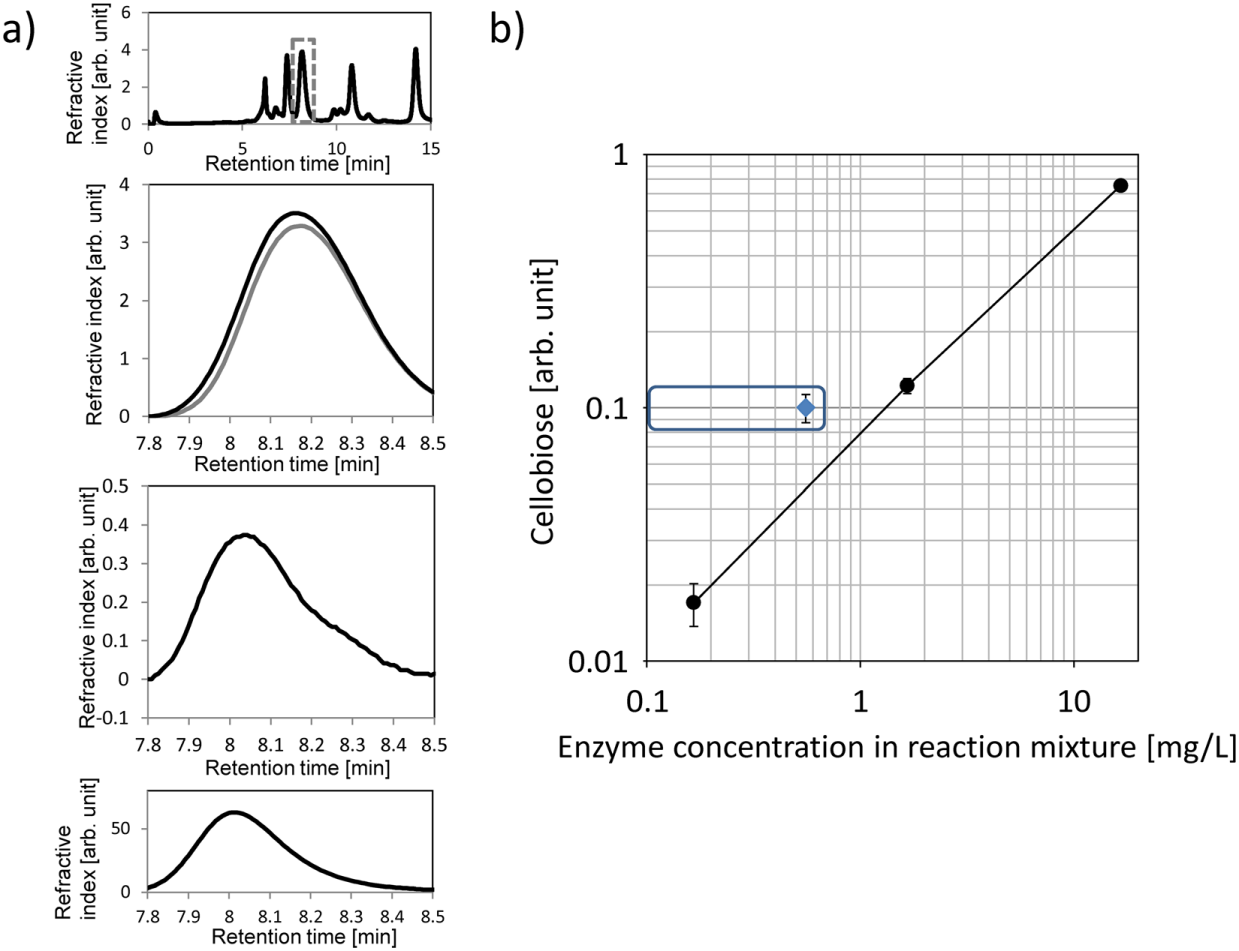
Hydrolytic activity assays for N62A mutant. (**a**) Chromatograms of reaction aliquots with N62A and cellobiose standard. Upper two charts show the retention time ranges 0–15 min and 7.8–8.5 min. Black line: reaction aliquot with substrate. Grey line: reaction aliquot without substrate. Third chart shows the difference in the two values (with and without substrate). Fourth chart shows the chromatogram of cellobiose, 0.15% (w/v). (**b**) Hydrolytic activity of WT at three different concentrations and the N62A mutant. Cellobiose refers to the HPLC peak area of cellobiose delivered by the action of *Tc*Cel7A in the activity assay. Black circles: WT. Blue diamond: N62A mutant, assuming a concentration below 0.5 mg/L in the reaction mixture. Error bar: Standard deviation.

First, we performed hydrolysis experiments for WT and the nine mutants using commercially available microcrystalline cellulose as the substrate. Cellobiose was detected as the product by HPLC (Fig. 4a, Supplementary Fig. S9). The specific weight activity was calculated for each enzyme species relative to WT by dividing the cellobiose amount by the enzyme amount (Table 1). The benchmark value of WT was 0.204 U/mg. Among the nine mutants, N62A and K203A exhibited significantly higher activities than WT. As the N62A concentration was at least 30 times lower than that of WT, we also examined 1/10 and 1/100 dilutions of WT (Fig. 4b). A comparison of N62A (measured) and WT (estimated) at the same concentration (<0.56 mg/L, stock concentration of below 5 mg/L divided by the dilution when preparing the reaction mixture) confirmed that N62A was at least twice as active as WT.

The hydrolytic activities of WT and the two mutants were also compared using different assay methods and on different substrates, including cellulose from Merck and pretreated corn stover. Data showed that N62A had more than two-fold greater activity than WT on all substrates (Table 2). The enhancement exhibited by N62A might include some overestimation due to the lower test concentration compared with that of WT, as discussed above.

**Table 2:** Hydrolytic activities of WT, N62A, and K203A from different culture batches on different substrates. Hydrolytic activity values from duplicated experiments are listed with standard deviations in parentheses.

Substrate	Assay	Activity			Culture no. of N62A
		WT	N62A	K203A	
Cellulose, Sigma (excerpted from Table 1)	HPLC	1.00 (0.01)	>3.85 (0.49)	1.26 (0.05)	1
Cellulose, Merck	HPLC	1.00 (0.04)	>4.57 (0.45)	1.36 (0.05)	
Cellulose, Merck	HPLC	1.00 (0.05)	>3.78 (0.90)	ND	2
Cellulose, Sigma	DNS	1.00 (0.42)	2.20 (1.12)	1.89 (0.58)	3
Pretreated corn stover	HPLC	1.00 (0.07)	>3.92 (0.34)	1.00 (0.15)	4

### Molecular dynamics (MD) simulation

**Figure 5:**
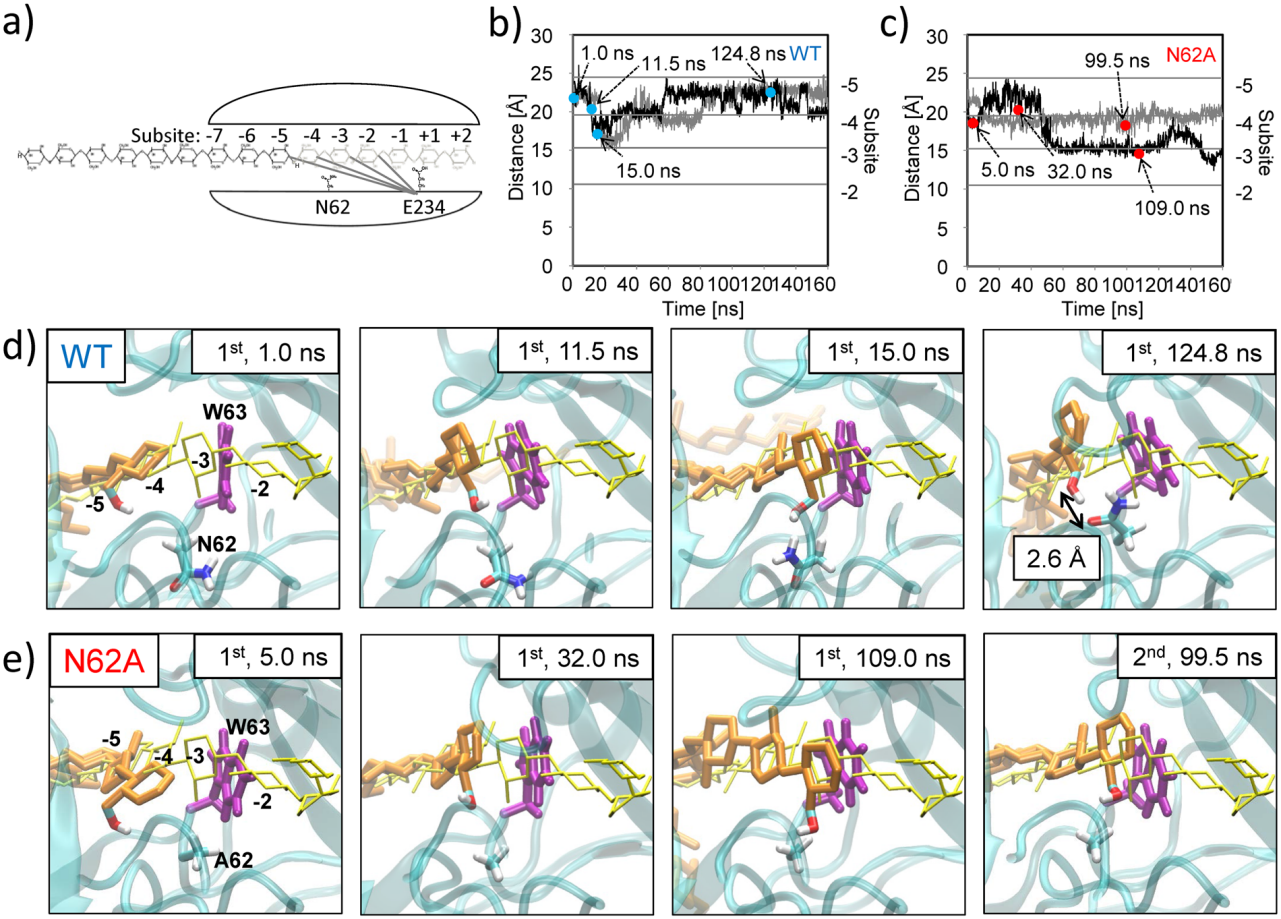
Substrate threading in MD simulations of WT and the N62A mutant. (**a**) Schematic diagram of the initial cellulose position (reducing end at subsite −5) and the distance between each subsite (−5 to −2) and catalytic residue E234. (**b**) Time evolution of distance between the C1 atom of the reducing end glucose and C_α_ atom of catalytic base E234 in the MD simulation of WT (first run in black, second run in grey), and (**c**) the same time evolution in the MD simulation of N62A (similar colours). The distances for each subsite (−5 to −2) in (a) are plotted with a line to scale the position of the cellulose substrate. Several stages are indicated by blue (WT) or red (N62A) circles. (**d**) Snapshots of substrate threading in the MD simulations of WT depicted at 1.0, 11.5, 15.0, and 124.8 ns from the first run correspond to initial (1.0 ns), pre-procession (11.5 ns), procession (15.0 ns), and recession (124.8 ns) stages, respectively. The threading cellulose chain is represented by its constituent carbon/oxygen ring chain (thick orange stick). In the leading glucosyl unit, the hydroxyl group at C6 is also drawn. To indicate the position of each subsite, the cellulose strand from the homology model (Fig. 1a) is superimposed (thin yellow stick of carbon ring). N62 and W63 sidechains are shown as thick sticks (CPK colouring and purple, respectively). (**e**) Substrate in the N62A mutant depicted at 5.0, 32.0, and 109.0 ns from the first run and 99.5 ns from the second run, corresponding to initial (1^st^, 5.0 ns), pre-procession (1^st^, 32.0 ns), and procession (1^st^, 109.0; 2^nd^, 99.5 ns) stages, respectively.

To elucidate the mechanism behind the activity enhancement afforded by replacing N62 with alanine, we conducted MD simulations of WT and the N62A mutant (two runs each, Fig. 5, Supplementary Movies 2 and 3). Starting from the initial position of the reducing end (cellononaose) at subsite −5 (Fig. 5a), we examined the processive movement of the cellulose strand into the catalytic tunnel. For motion analysis, we measured the distance between the C1 atom of the reducing end glucose and C_α_ atom of E234 at the catalytic site (Fig. 5b,c). Snapshots at four different stages along the time course are shown in Fig. 5d. For both runs using WT, the cellulose strand had almost proceeded to subsite −3 after around 15 ns. Notably, when the reducing end glucose had proceeded to subsite −4 (1^st^, 11.5 ns), it was twisted and aligned along W63, where the two ring planes were almost parallel with a distance of about 4 Å, suggesting a weak hydrophobic stacking interaction^27^. After the cellulose strand reached subsite −3 (1^st^, 15.0 ns), it was observed to move backwards, finally fluctuating between subsites −4 and −5 (1^st^, 124.8 ns). In this state, the hydroxyl group at C6 in the leading glucosyl unit and the sidechain of N62 were in close proximity (distance between hydroxyl group oxygen atom and N62 oxygen atom was 2.6 Å; for the time course, see Supplementary Fig. 10a), suggesting a tight hydrogen bond. On the other hand, in one run using N62A (Fig. 5e), we observed twisting of the reducing end glucose in association with W63 (1^st^, 32.0 ns), but no apparent recession of the cellulose strand. The cellulose strand proceeded smoothly toward subsite −3 and stayed there (1^st^, 109.0 ns). In the other run, the distance stayed at around −4 during the time course. However, close examination of the typical conformation of the reducing end glucose (2^nd^, 99.5 ns) showed that it was almost identical to that of the cellulose staying at subsite −3 in the previous run (1^st^, 109.0 ns).

**Figure 6:**
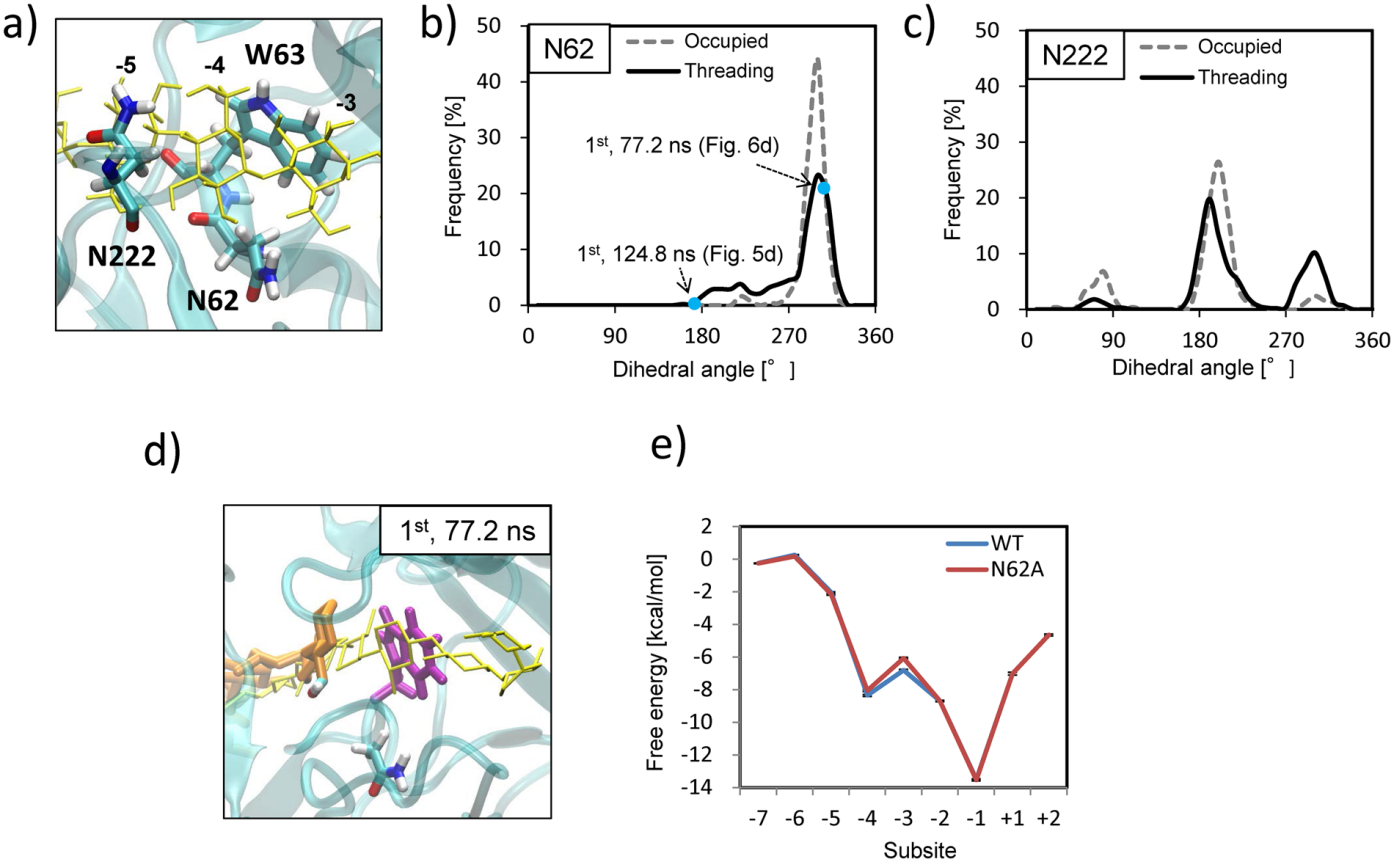
Comparison of the interactions of N62 and subsite −4 with that of N222 and subsite −4 in WT simulations. (**a**) Subsites −3 to −5 and three residues near the location: N62, W63, and N222. (**b**) Histograms of the dihedral angle (N–C_α_–C_β_–C_γ_) of N62 in substrate threading (black solid) and occupied (dashed grey) simulations, and (**c**) the same histograms for N222 (similar colours). (**d**) Typical conformation of the major frequency with a dihedral angle of 309° (approx. 300°) for N62 (1^st^, 77.2 ns). (**e**) Free energy landscapes of substrate threading in WT and the N62A mutant. Binding free energy of the cellulose substrate with WT (blue) or N62A (red) mutant at each subsite was calculated using the MM/GBSA method. Error bar: Standard error.

Next, we compared the interactions of N62 and subsite −4 with that of N222 and subsite −4 in WT simulations. Notably, the two asparagine residues interacted with subsite −4 (Fig. 6a). For the respective asparagine residue, histograms of the dihedral angle formed by N–C_α_–C_β_–C_γ_ in “threading” (Fig. 5b, 60–160 ns trajectory in the first run (black line); for the time course, see Supplementary Fig. 10b) or “occupied” (Fig. 1b) cases are shown in Fig. 6b,c. N62 adopted almost the same conformation, with a dihedral angle of approx. 300° (main frequency, >45%), in the simulation for the occupied case. In contrast, in the threading case, a similar conformation had a frequency of 25% (major, but less than in the occupied case, see Fig. 6d for a typical conformation), while the twisted conformation with dihedral angles <220° made a significant distribution (minor) that was not observed in the occupied case. This conformation was favourable for hydrogen bonding with the hydroxyl group at C6 of the leading glucosyl unit positioned between subsites −4 and −5, as previously shown in Fig. 5d, at 124.8 ns (dihedral angle = 174°). In the N222 reference, the positions and shapes of the three peaks were almost identical in the occupied and threading runs, although some changes in the frequency distribution among the three peaks were observed.

Moreover, to explore the free energy landscapes of substrate threading in WT and the N62A mutant, we calculated the binding free energies of the cellulose substrate with WT or N62A at each subsite using the molecular mechanics/generalized Born surface area (MM/GBSA) method using the trajectories of the occupied simulations (Fig. 6e). The landscapes shared a common funnel-like topology that had a global minimum point at subsite −1 (catalytic site), a local minimum point at subsite −4 (N62 hydrogen bonding), and a local maximum point at subsite −3. At the local maximum point (subsite −3), the binding free energy for N62A was slightly weaker than that for WT (by 0.75 kcal/mol), which was attributed to the lack of hydrogen bonds with the N62 residue.

## Discussion

In this study, to investigate the structural features of the catalytic tunnel of GH7 CBHs, homology modelling of *Tc*Cel7A was conducted using *Tr*Cel7A as a template (Fig. 1a, Supplementary Fig. S2, Supplementary Movie 1). The amino acid sequence and resultant structure of *Tc*Cel7A were compared with those of *Tr*Cel7A and *Pc*Cel7D (Supplementary Figs S1 and S2), showing that the three proteins shared similar primary and tertiary structures. In addition to the structure of the protein, the cellulose substrate had a characteristic conformation when complexed with GH7 CBHs. In the subsequent MD simulation, the model structure of *Tr*Cel7A was confirmed to be very stable, as evaluated using the RMSD (Fig. 1b) and the distances between each subunit of the cellulose substrate and the catalytic residue (E234) (Fig. 1c). Moreover, we confirmed the constancy of cellulose chain conformation, especially at subsites −3 to −5 (Supplementary Fig. S3).

By employing *A. oryzae* as an expression host, we succeeded in producing mutants, except for tryptophan-to-alanine mutants, namely W63A and W65A (Fig. 3). Previously, Nakamura *et al*.^32^ obtained the corresponding W65A mutant of *Tr*Cel7A (W40A) by employing *T. reesei* as the expression host. Moreover, Kari *et al*.^33^ obtained the corresponding W63A mutant of *Tr*Cel7A (W38A) by employing *A. oryzae* as the expression host. Although the reason for failed expression of these two mutants of *Tc*Cel7A in the present system was not clear, a possible explanation was that W63 and W65 are indispensable in maintaining the molecular structure of *Tc*Cel7A. Whether the failure to produce these alanine-substituted mutants depended on the synthetic genes and/or expression host would be resolved via expression studies of *Tr*Cel7A mutants in *A. oryzae* and/or of *Tc*Cel7A mutants in *T. reesei*. We found that *Tc*Cel7As recombinantly expressed by *A. oryzae* was highly *N*-glycosylated. This *N*-glycosylation did not affect the weight specific activity for WT. However, its effects on the productivity or stability of recombinantly expressed *Tc*Cel7A are yet to be studied. As some mutant proteins were unstable in ultrafiltration (centrifugal concentration), we decided to characterise the mutant proteins only as the culture supernatant of the production host strain without concentration or purification. Other purification/concentration methods, such as employing affinity tags, require testing to further examine the reaction kinetics with *Tc*Cel7A WT and mutants.

High CBH loading on cellulose substrate is known to lead to substrate saturation, where the activity is not proportional to enzyme loading^41^. In this study, the substrate concentration was 4% (w/v) and the enzyme loading was around 10 mg/L or less (Fig. 4b), which corresponded to less than 1 μM. This enzyme loading was in the range where activity is proportional based on the *Tr*Cel7A data^41^. The activity data obtained with *Tc*Cel7A WT and its 1/10 and 1/100 dilutions confirmed this assumption (Fig. 4b). More extensive assays will be considered in future investigations, preferably using purified WT and mutants, to study the dependence of activity on enzyme loading and more precisely determine the weight specific activity enhancement ascribed to the amino acid mutation.

The Avicel assay of the alanine mutants (Table 1) showed that mutations at the Entrance or Loop moderately decreased the activity, except for N125A, in which the activity was unaffected, and W65A (not detected), while those at the Base increased the activity, except for W63A (not detected). The decreased activity in the former case could be explained by the conventional knowledge that a processive enzyme might bind its polymeric substrate with moderate affinity at multiple adjacent sites distributed within a catalytic tunnel^22^. Therefore, the contribution of the processive mechanism from each interacting residue was relatively weak, with one alanine mutation not leading to a crucial loss in cellulase activity. In contrast, the increased activity in the latter case was somewhat unexpected. Among the two mutants possessing significantly higher activity than that of WT toward microcrystalline cellulose, the N62A mutant showed higher activity than WT toward pretreated corn stover, while the enhanced activity of the K203A mutant was diminished toward this natural biomass. It could be postulated that the enhanced activity of the K203A mutant toward reagent cellulose was due to a lower substrate affinity, which is supposedly prominent when the specific cellulose surface area accessible to the enzyme is larger^33^.

To elucidate the mechanisms underlying the activity enhancement in the N62A mutant, we performed MD simulations of initial threading, which demonstrated distinctive time-evolving trajectories for WT and N62A (Fig. 5, Supplementary Movies 2 and 3). The most prominent finding was that the leading glucosyl unit deviated from the correct path and was sterically hindered from moving further by N62 (Fig. 5d, 1^st^, 124.8 ns). The histogram of the dihedral angle of N62 during threading (Fig. 6b) showed that this conformation was unique to “threading” and not seen in the “occupied” cases. N62 appeared to play a role in pulling the cellulose substrate into the catalytic tunnel by adopting the conformation with a smaller dihedral angle, which could have a positive effect on processive movement. However, this temporary hydrogen bonding also tended to deviate the cellulose substrate from the correct path, as described above. Our activity assay demonstrated that this negative effect outweighed the positive effect, which was related to recession of the threading. Substitution of N62 with alanine resulted in a smoother surface inside the catalytic tunnel with no protruding and interacting residues, which led to a higher activity. In the reference case, N222 did not exhibit any characteristic conformation associated with substrate threading. Two MD simulation runs were performed for N222A, in which no processive movements of the substrate cellulose were observed (Supplementary Fig. S11).

When the leading glucosyl unit had proceeded to subsite −4 in the MD simulations, W63 was found to facilitate twisting of the leading glucose, possibly due to a weak hydrophobic stacking interaction, so that the leading glucose could easily proceed to subsite −3 (Fig. 5d, 1^st^, 11.5 ns). Although the crystal structure suggests that the W63 homologue interacts with the glucose at subsite −4 of the cellononaose complexed with *Tr*Cel7A^27^, the major twisting of the cellulose chain at subsite −4/−3 was not ascribed to this interaction. To our knowledge, our results are the first demonstration of how W63 functions dynamically as the substrate proceeds through the catalytic tunnel. When the leading glucosyl unit was twisted at subsite −4, its C6 moiety was situated on the opposite side to cellononaose in the homology model and was, therefore, in close proximity to N62, resulting in hydrogen bonding (Fig. 5d, 1^st^, 124.8 ns). N62 was likely to have a similar effect on the successive threading of cellobiose per cycle. In a trial MD simulation, starting from an initial configuration in which subsites −5 to −1 were occupied with a cellulose strand and subsites + 1 and + 2 were empty, no significant substrate movement along the catalytic tunnel was observed within the calculation time of 160 ns.

The W38A mutant of *Tr*Cel7A (corresponding to the W63A mutant of *Tc*Cel7A) has been shown to produce a two-fold increase in the maximum quasi-steady-state hydrolytic rate in a certain range of substrate and enzyme loading, suggested to be caused by reduced substrate affinity^33^. More comprehensive data are required to better understand the function of W38.

The free energy landscape of substrate threading is shown in Fig. 6e, in which the energy contributions are resolved for each glucosyl unit. The landscape shape was funnel-like, with the global minimum at subsite −1. This seemed reasonable for the enzyme to repeatedly perform the cycle of substrate chain threading, hydrolysis, and cellobiose expulsion. Hydrolysis took place at subsite −1/ + 1, at which point the substrate was rigidly fixed to the correct coordinate by interactions with the surrounding residues. This was in good accordance with previous findings that the fluctuation of the glucosyl unit at subsite −1 was smaller than those at the entrance (subsites −7 and −6) or end (subsite + 2) of the catalytic tunnel, as demonstrated in the MD simulations of several GH7 CBHs from different organisms^28,42^. Notably, at subsite −3, the binding energy reached a local maximum, suggesting a loose interaction between the substrate and enzyme in that vicinity. At subsite −3, the binding free energy of N62A was weaker than that of WT by 0.75 kcal/mol, roughly equivalent to the energy of one hydrogen bond (0.5–1.8 kcal/mol)^43^. Colussi *et al*.^29^ conducted calorimetric experiments to study the interactions of cello-oligosaccharides with a catalytically deficient mutant (E212Q) of *Tr*Cel7A, and suggested that filling subsites −2 and −1 had no, or a slightly unfavourable, impact on the net affinity (*ΔG°*), despite these subsites being rich in enzyme-ligand hydrogen bonding. Therefore, these stabilising bonds were compensated by unfavourable steric effects, including cellulose strand twisting or pyranose ring distortion. Substrate twisting was not explained by the static image of interaction energy and, therefore, might be more accurately interpreted as a dynamic phenomenon, including the contribution of the temporal action of the surrounding residues, such as N62 and W63 herein. The loose and appropriate interaction between the glucosyl unit and enzyme residues near subsite −3 would allow the cellulose strand to have a flexible conformation, leading to twisting and rapid threading. Further investigation of the twisting around subsite −4/−3 is required.

Improved cellulase activity has a multitude of impacts on the entire biofuel production process, which comprises pretreatment, enzymatic hydrolysis, fermentation, and condensation (distillation and molecular sieving)^15,44^, and would reduce production costs significantly. Our results demonstrate the potential of site-directed mutagenesis technology to enhance cellulase activities^45^. The N62A mutant was much less productive with our *A. oryzae* host and unstable in physical processing, such as ultrafiltration, which are major drawbacks to commercial application. N62 is conserved in GH7 CBH across various microorganisms, including *T. reesei* and *P. chrysosporium* (Supplementary Fig. S1). This might suggest that this residue plays an essential role in stabilising the molecular structure. Experiments to verify whether replacing the corresponding asparagine residues with alanines is effective in GH7 CBHs from other microorganisms are warranted. Furthermore, a saturation mutagenesis method could detect another amino acid that gives a similarly increased activity to N62A without the commercial drawbacks. These findings would yield valuable information and provide further systematic analysis of the numerous diverse biorefinery applications.

## Methods

### Construction of *Tc*Cel7A encoding plasmids and transformation

An Applied Biosystems thermal cycler and KOD Plus polymerase (Toyobo) were used for all PCR experiments. The PCR reaction mixture and conditions for the polymerase were as described in the user manual. The original gene was amplified from the genomic DNA of *T. cellulolyticus* strain H1^8^ by PCR (sequence: GenBank Accession Number E39854). The expression vector was constructed by integrating the *enoA* d4 promoter^46^, *agdA* terminator, and *pyrG* genes from *A. oryzae* on pMD20 (Takara). The amplified *Tc*Cel7A gene was inserted between the promotor and terminator regions. For alanine scanning experiments^47^, point-mutated genes were prepared using an In-Fusion HD Cloning Kit (Clontech Laboratories, Inc.) with synthetic PCR primers possessing site-directed mutated bases (nine mutants: N62A, W63A, W65A, S72A, N74A, N125A, K203A, N222A, and T223A). *Aspergillus oryzae* strain NS4DLP, which lacks the *pyrG* gene and, therefore, requires uridine in the culture medium for growth, was used as the enzyme production host^48–50^. Transformation was performed as described previously^46^. *A. oryzae* cultures were maintained using medium containing 2% (w/v) dextrin hydrate, 1% (w/v) peptone, 0.1% (w/v) casamino acid, 0.5% (w/v) KH_2_PO_4_, 0.1% (w/v) NaNO_3_, and 0.05% (w/v) MnSO_4_ **·**4H_2_O. The pH was adjusted to 6.0 using NaOH.

### Enzyme production

The medium used contained 10% (w/v) molasses, 2.5% (w/v) peptone, 2.4% (w/v) KH_2_PO_4_, and 0.12% (w/v) MgSO_4_, with the pH adjusted to 6.0 using NaOH. *A. oryzae* transformants were grown in the culture medium (10 mL) at 30 °C for 3 days. The culture broth was filtered through a 0.22-μm Durapore membrane (Millipore). Production of the target enzymes contained in the filtrates was assayed by SDS-PAGE. Precast gels, and preformed running and CBB staining buffer solutions, were obtained from Atto Corporation. Prestained protein standard was purchased from Bio-Rad. Quantification of corresponding bands on the SDS-PAGE gel image was performed using a Bio-Rad imager to estimate the target enzyme concentrations^51^. Bovine serum albumin (BSA, Nacalai Tesque) was employed as the calibration standard. Pseudo-colour was applied using Image Lab (Bio-Rad). Calibration for the quantification of each target protein was conducted using the same software. For N62A mutant (Culture 3), see Supplementary Fig. S8. Production of WT and the four mutants, N62A, W63A, W65A, and K203A, was examined by western blotting using a specific rabbit polyclonal WT antibody as the primary antibody and the ECL prime kit including a horseradish peroxidase (HRP)-conjugated antibody (GE Healthcare). The assay was performed following manufacturer instructions. Ultrafiltration of the expressed proteins was performed using a commercially available centrifugal concentrator (MWCO: 3,000; Vivaspin, Sartorius) at 4 °C following manufacturer instructions. Deglycosylation of recombinantly produced *Tc*Cel7A was performed by reacting PNGase F (New England BioLabs) with the culture supernatant sample according to manufacturer instructions.

### Biomass pretreatment

Corn stover pretreated with dilute acid was used to assess the activity of WT and alanine-substituted mutants^52–54^. Dry corn stover was ground, passed through a 3-mm mesh, and mixed with twice its weight of aqueous sulfuric acid (2.5%, w/v). The mixture was kept at 150 °C for 10 min and then neutralised with NaOH. The pretreated biomass was then dried at 50 °C for 1 day and kept dry (water content, 7%) in a desiccator until being used in downstream activity assays.

### Activity assay

Hydrolytic assays were carried out by incubating mixed solutions of each *A. oryzae* transformant (20 μL), 4% (w/v) reagent cellulose (100 μL; Avicel PH-101 from Sigma or microcrystalline cellulose from Merck, cat. no. 102331) or pretreated corn stover, and 0.2 M acetate buffer (pH 5.5; 60 μL) at 50 °C for 1 h. Controls were prepared in the same way, but without substrate solids. Hydrolysis experiments were performed in duplicate. Aliquots after incubation were centrifuged (5,000 × g), and the supernatants were assayed for cellobiose by HPLC using an HPX87 P column (Bio-rad) and RI detector (Waters)^55^. Areas were calculated for the retention time range 7.8–8.5 min. Differences ascribed to the action of the target enzyme were obtained by subtracting the results without substrates from those with substrates (Supplementary Fig. S9). Hydrolysis activity toward Avicel was evaluated by colourimetry using dinitrosalicylic acid (DNS)^56,57^. DNS solution was prepared from two solutions, KNaC_4_H_4_O_6_·4H_2_O (Rochelle salt; 300 g) in 2 M NaOH aqueous solution (200 mL) and DNS (10 g) in purified water (400 mL; Milli-Q), which were mixed and diluted with purified water to a final volume of 1 L. To assay the reducing sugar concentration, we added the same amount of DNS solution to aliquots from reaction mixtures before and after incubation, heated these solutions at 100 °C for 5 min, and then measured their absorbance at 540 nm. We obtained the differences ascribed to the action of the target enzyme by subtracting the results without substrates from those with substrates. Hydrolysis activities toward CMC and pNPG were assayed according to the protocols of Kansarn^58^, except that DNS colourimetry was employed to evaluate CMC saccharification. CMC (cat. no. C5678) and pNPG (N1627) reagents were purchased from Sigma.

### Homology modelling

The primary sequence of the catalytic domain of *Tc*Cel7A was modified by trimming the N-terminal signal peptide (1–25 aa) and the C-terminus (461–529 aa) for modelling (Supplementary Fig. S1). A structural model for the catalytic domain was then built using *Tr*Cel7A (PDB:8CEL^20^) as a template using the Discovery Studio 4.0 (Accelrys) software package. During energy minimisation to refine the positions of all heavy atoms in the protein, the structure of the substrate glycan in the template *Tr*Cel7A was employed and fixed. The N62A mutant was created by replacing the asparagine residue with alanine *in silico*. Protonation states at pH 7.0 were determined using the PDB2PQR server^59^, and nine disulfide bonds were assigned according to the template molecules. RMSD between the homology model and other GH7 structures were calculated using the RMSD Calculator plugin in VMD^60^.

### MD simulation

To simulate initial threading of the cellulose substrate for WT and the N62A and N222A mutants of *Tc*Cel7A, the reducing end glucose (head) of the cellulose strand (cellononaose) was initially positioned at subsite −5, and other parts were created automatically (“threading” case). The systems were fully solvated with explicit solvent, and 18 Na^+^ counterions were added to obtain electrostatic neutrality. All simulations were performed using AMBER14 MD software^61^. We employed the AMBER ff03 force field for proteins, the GLYCAM 06 force field for the cellulose strand, and the TIP3P model for water molecules. The systems were energetically minimised for the 300 steepest descent steps and equilibrated for 1 ns, with gradually reducing restraints. Finally, two 160-ns production runs were performed with different initial velocities for each system using a protocol similar to that described by Nakamura *et al*.^32^. Trajectory analysis was conducted using the AMBER module cpptraj and snapshot structures were visualised with VMD. An additional MD simulation was conducted for WT, in which a cellulose substrate (cellononaose) was situated to occupy all subsites (from −7 to + 2) initially (“occupied” case). Force fields and protocols were the same as described above, and the production run was performed for 25 ns. Binding free energies between *Tc*Cel7A and the glucosyl units positioned at each subsite were calculated using the MM/GBSA method^62^, which was integrated in the AMBER14 software.

## Electronic supplementary material

Supplementary Figures

Supplementary Movie 1

Supplementary Movie 2

Supplementary Movie 3
