# Supplementary material for: Alanine substitution in cellobiohydrolase provides new insights into substrate threading
Source: Sci Rep. 2017 Nov 24;7:16320. doi: 10.1038/s41598-017-16434-x (PMC5701224; doi:10.1038/s41598-017-16434-x)
Supplement: Supplementary file 1 — Supplementary Figures [file 41598_2017_16434_MOESM1_ESM.pdf]

Title:

Alanine substitution in cellobiohydrolase provides new insights into substrate threading

Authors:
Shigenobu Mitsuzawa[1*],
Maiko Fukuura[1],
Satoru Shinkawa[1],
Keiichi Kimura[2],
and Tadaomi Furuta[3]

[1] Honda Research Institute Japan Co., Ltd., 8-1 Honcho, Wako-shi, Saitama, 351-0188, Japan
[2]Automobile R&D Center, Honda R&D Co., Ltd., 1-4-1 Chuo, Wako-shi, Saitama, 351-0113, Japan
[3]School of Life Science and Technology, Tokyo Institute of Technology, B-62 4259 Nagatsuta-cho, Midori-ku, Yokohama 226-8501, Japan

Corresponding author:
*Shigenobu Mitsuzawa

Honda Research Institute Japan Co., Ltd., 8-1 Honcho, Wako-shi, Saitama 351-0188, Japan
Tel: +81-80-4919-2650
E-mail: shigenobu.mitsuzawa@jp.honda-ri.com

SUPPLEMENTARY FIGURE S1. Multiple sequence alignment of three GH7 CBH enzymes: *Tc*Cel7A, *Tr*Cel7A, and *Pc*Cel7C. Sequences are available from GenBank or PDB: *Tc*Cel7A (GenBank: GAM33347), *Tr*Cel7A (PDB: 8CEL), and *Pc*Cel7D (PDB:1GPI). Alignments were constructed using Discovery Studio 4.0 (Accelrys). Amino acid residues are coloured based on four types: Identical, strongly similar, weakly similar, and other, with a colour gradient from dark green to white, respectively.

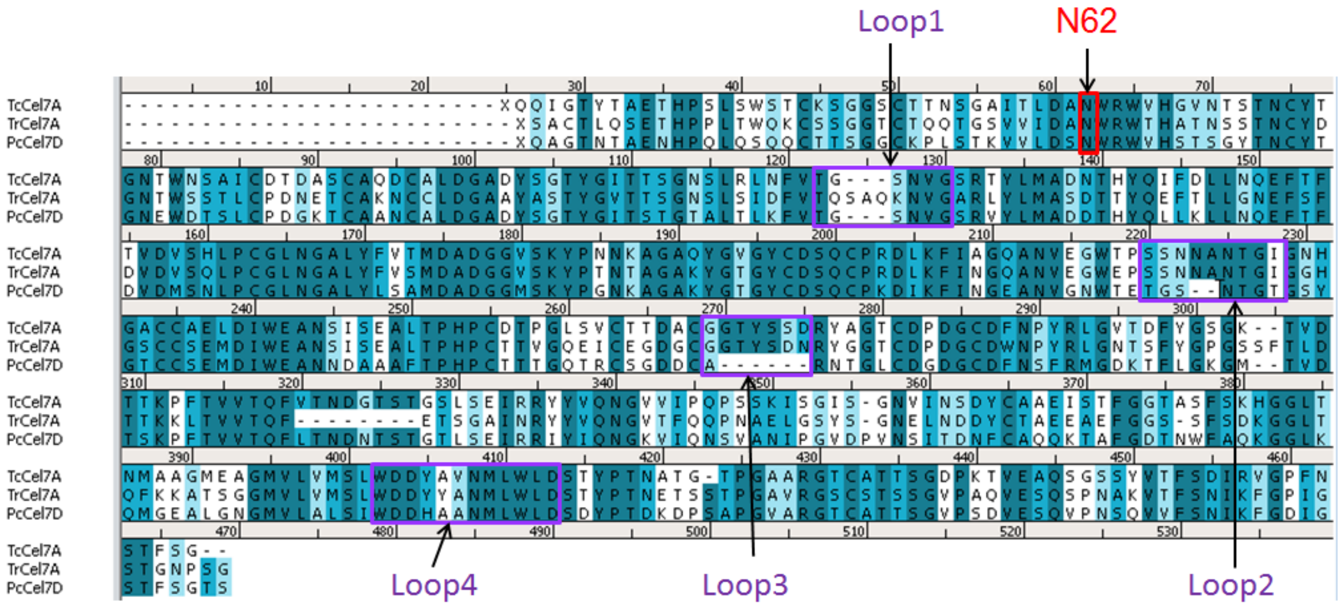

*Tc*Cel7A vs *Tr*Cel7A
Identity: 280 / 434 (64%)
Similarity: 391 / 434 (90%)

*Tc*Cel7A vs *Pc*Cel7D
Identity: 257 / 436 (58%)
Similarity: 390 / 436 (89%)

SUPPLEMENTARY FIGURE S2. Comparison of three GH7 CBH enzyme structures: *Tc*Cel7A, *Tr*Cel7A, and *Pc*Cel7C.

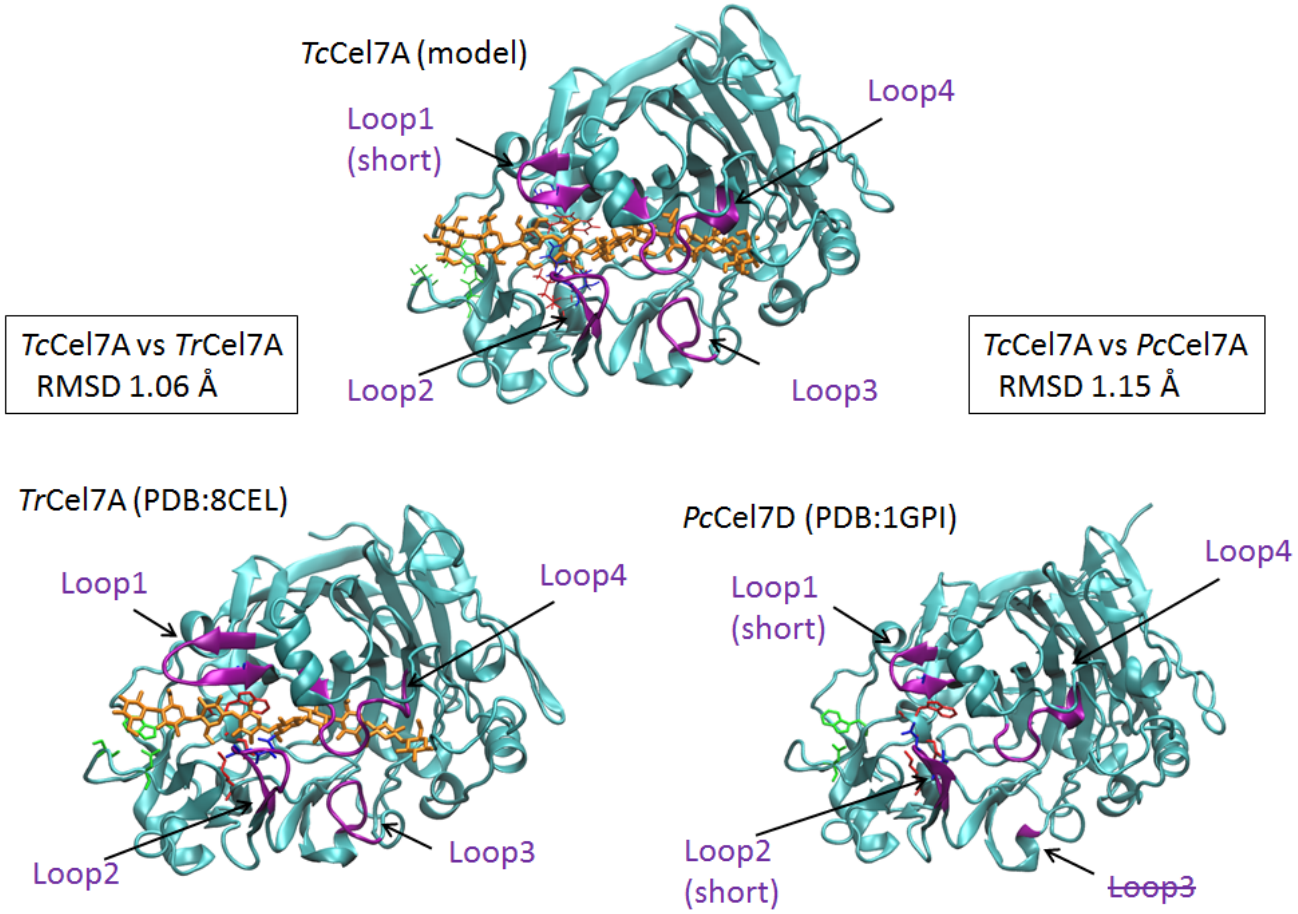

SUPPLEMENTARY FIGURE S3. Time evolution of the cellulose strand complexed with *Tc*Cel7A in a MD simulation.

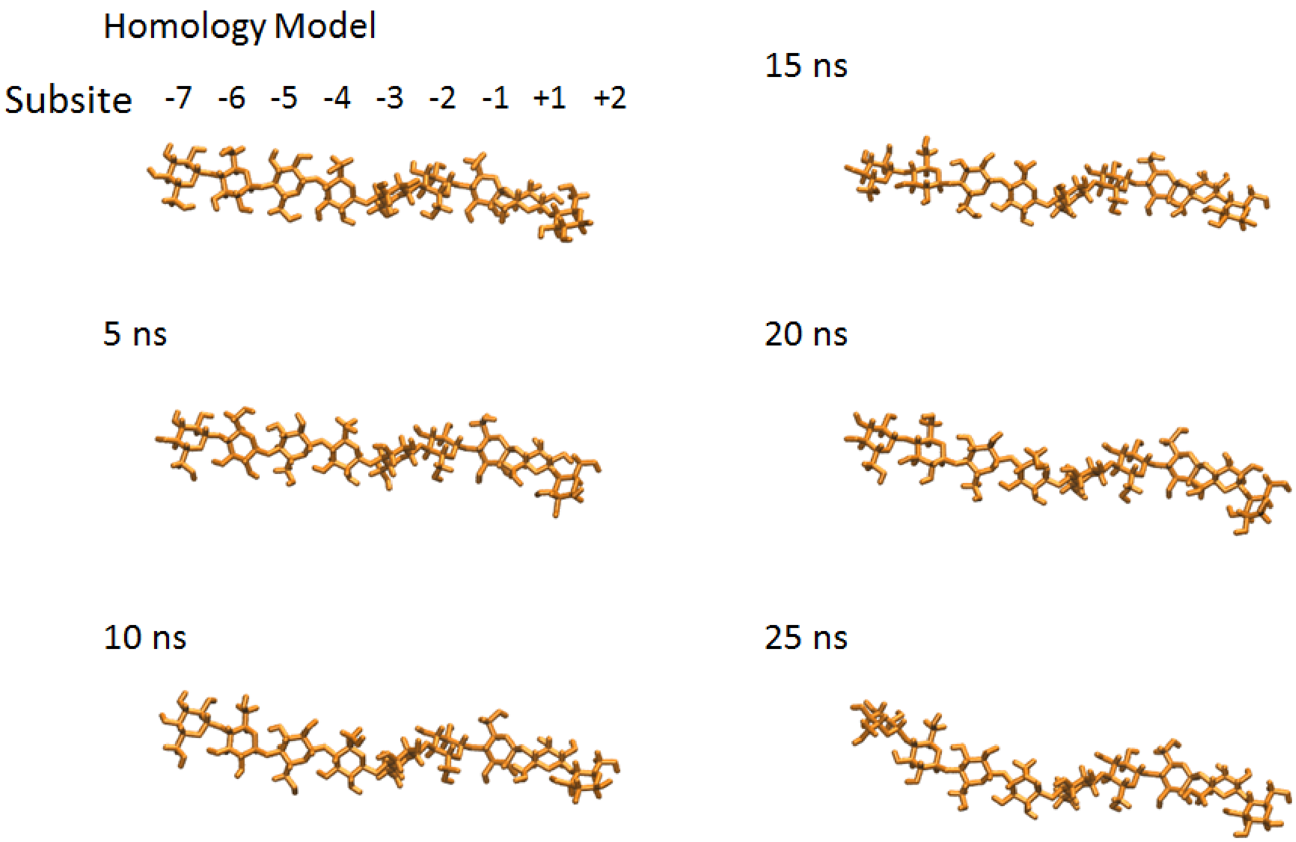

SUPPLEMENTARY FIGURE S4. Full-length gels shown in Fig. 3a.

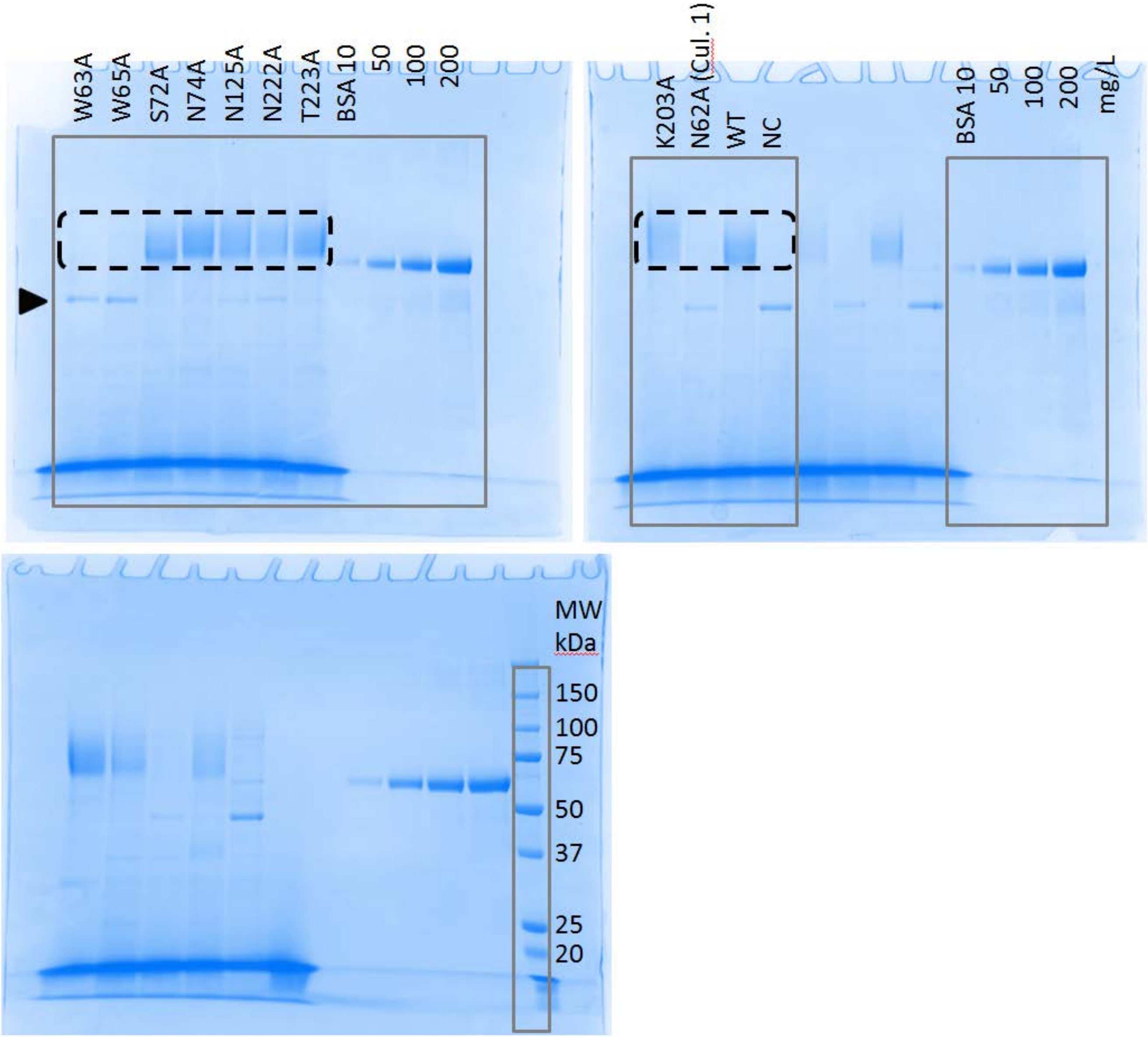

SUPPLEMENTARY FIGURE S5. Comparison of two *Tc*Cel7As. (Left) Purification from the culture supernatant of *T. cellulolyticus* by column chromatography. M: marker, 1: culture supernatant of *T. cellulolyticus*, 2: purified *Tc*Cel7A. Column chromatography was conducted using Toyopearl QAE-550C and Butyl-650M resins (Tosoh Bioscience). (Right) Recombinant production by *A. oryzae*. M: marker, 3: culture supernatant of *A. oryzae host.* 4: culture supernatant treated with PNGase F.

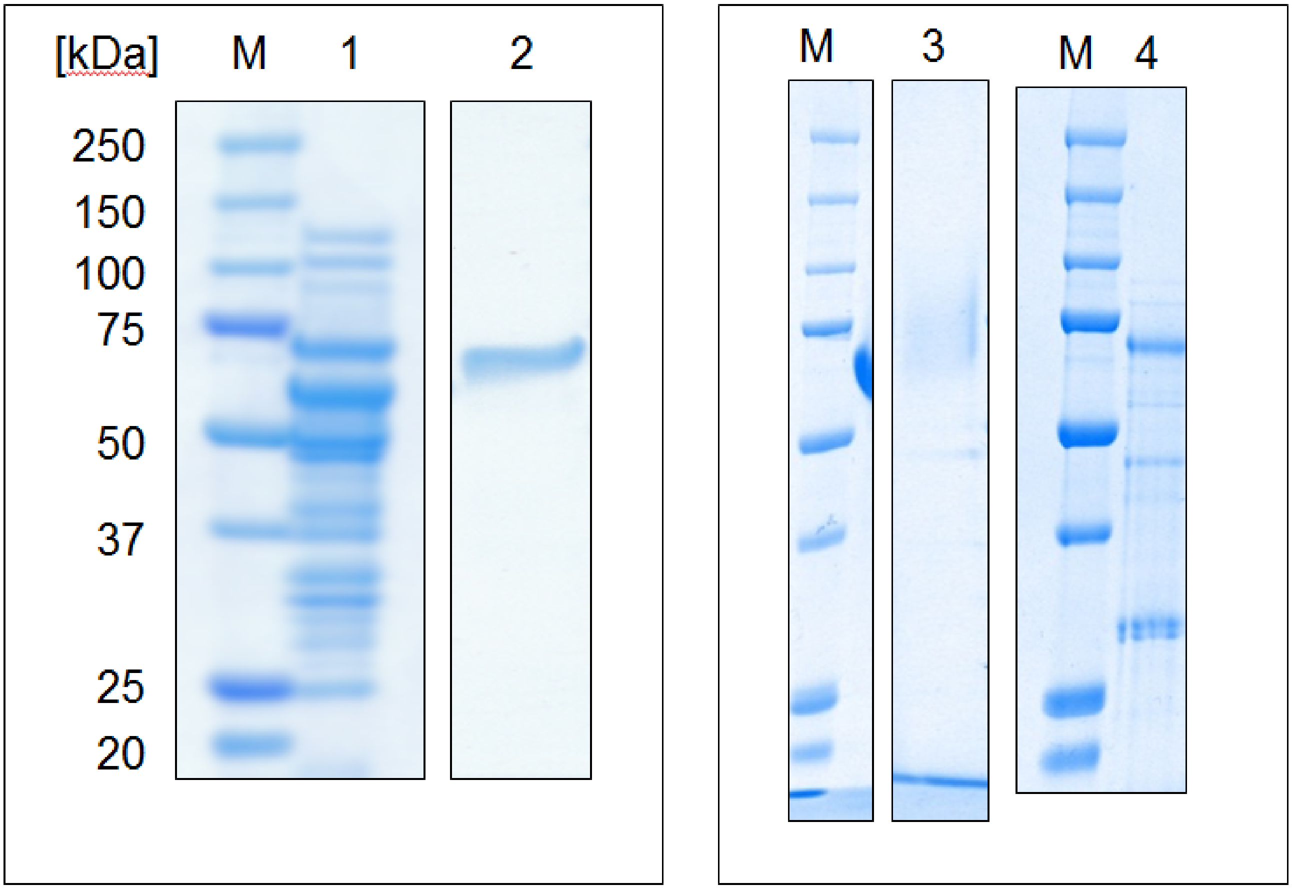

SUPPLEMENTARY FIGURE S6. Full-length blotted membrane shown in Fig. 3b.

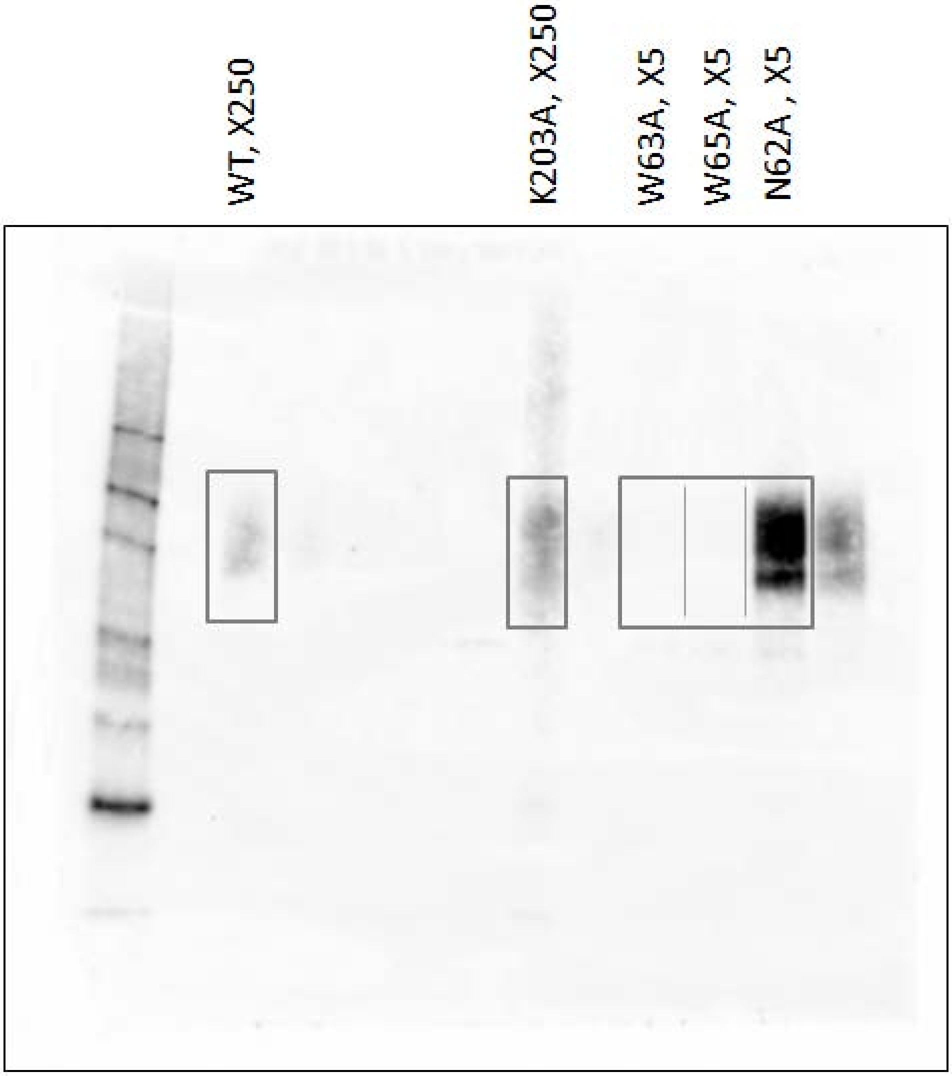

SUPPLEMENTARY FIGURE S7. Full-length gels shown in Fig. 3c with original colour.

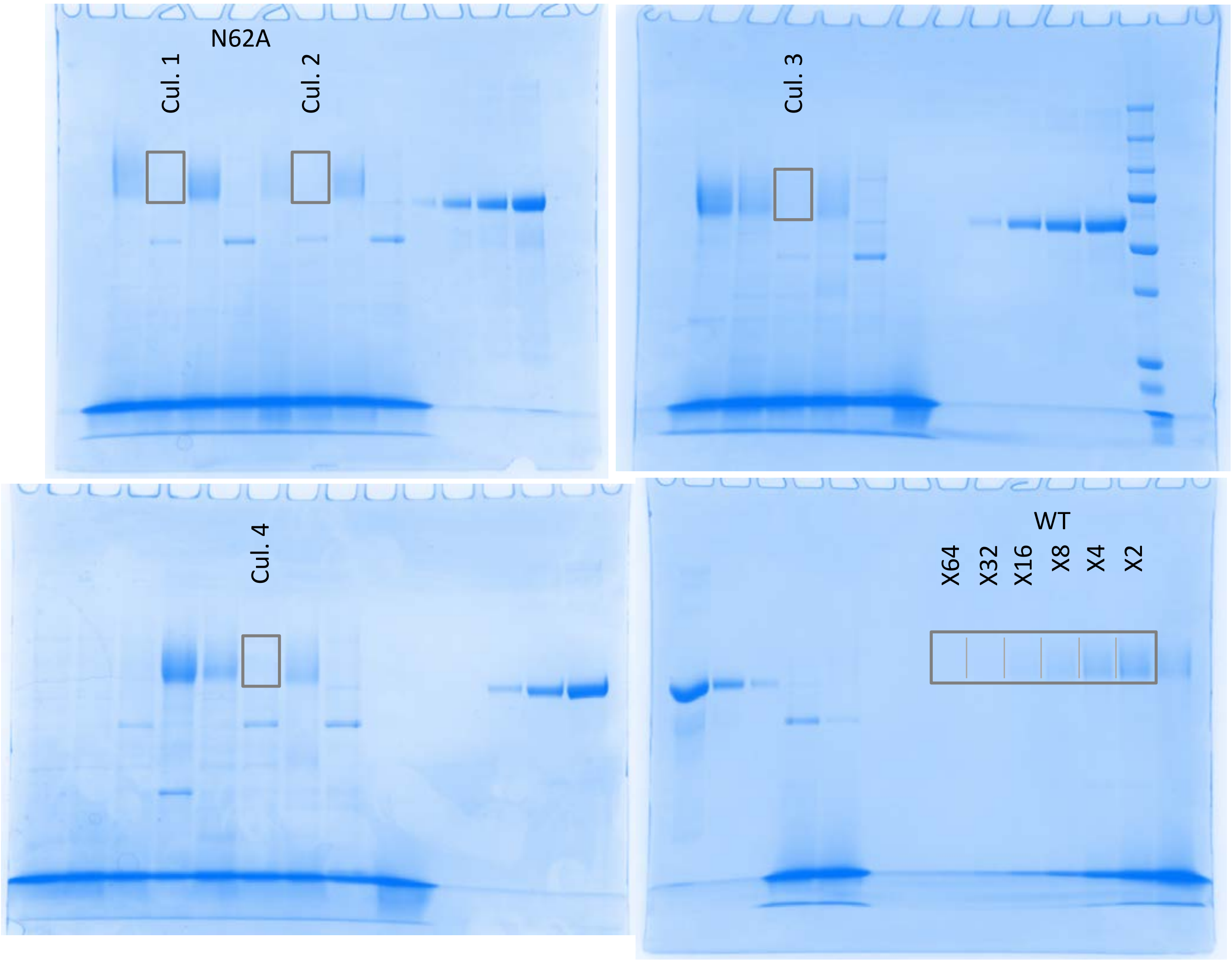

SUPPLEMENTARY FIGURE S8. Calibration for quantification of N62A mutant.

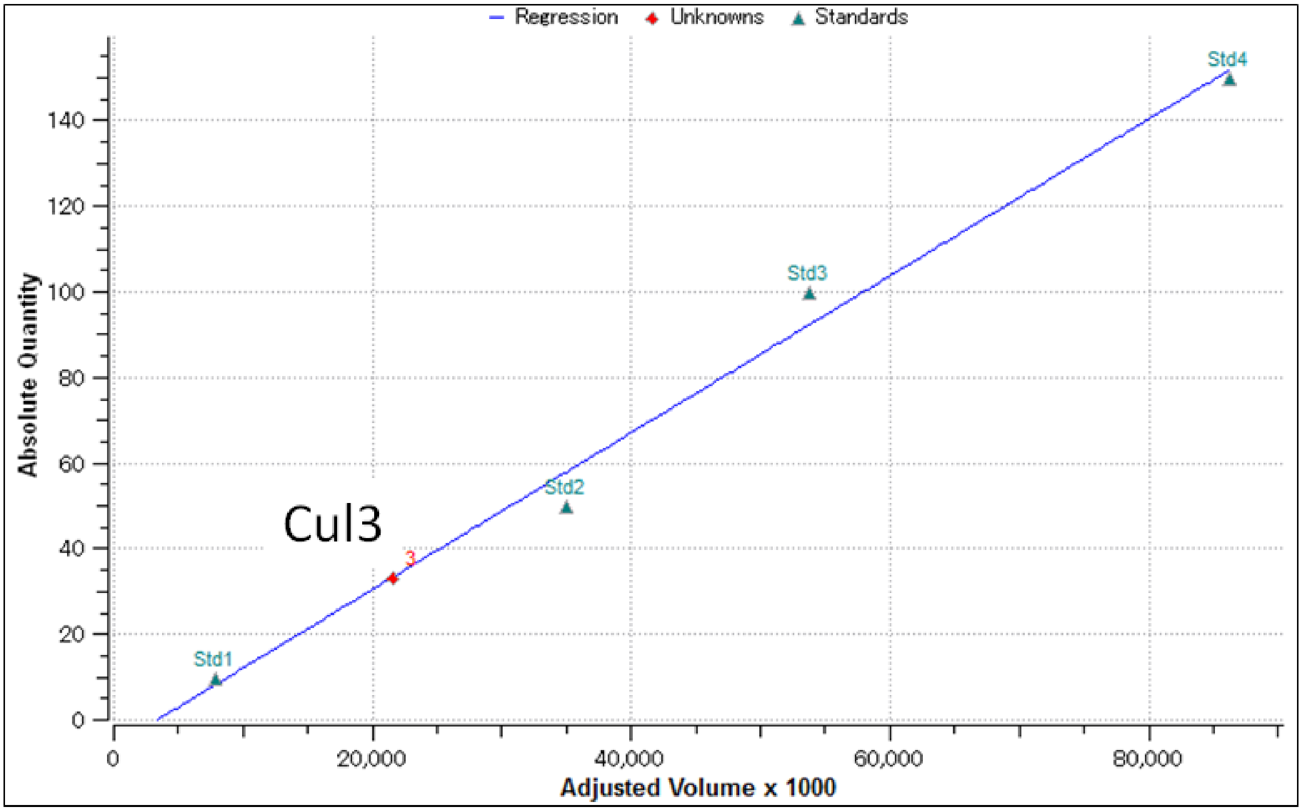

SUPPLEMENTARY FIGURE S9. Chromatograms of reaction aliquots. (a) 1/10 dilution of WT. The upper chart shows the retention time ranges 7.8–8.5 min. Black line: reaction aliquot with substrate. Grey line: reaction aliquot without substrate. The lower chart shows the difference between the two values (with and without substrate). (b) Culture supernatant of the untransformed strain (negative control, NC). (c) K203A mutant.

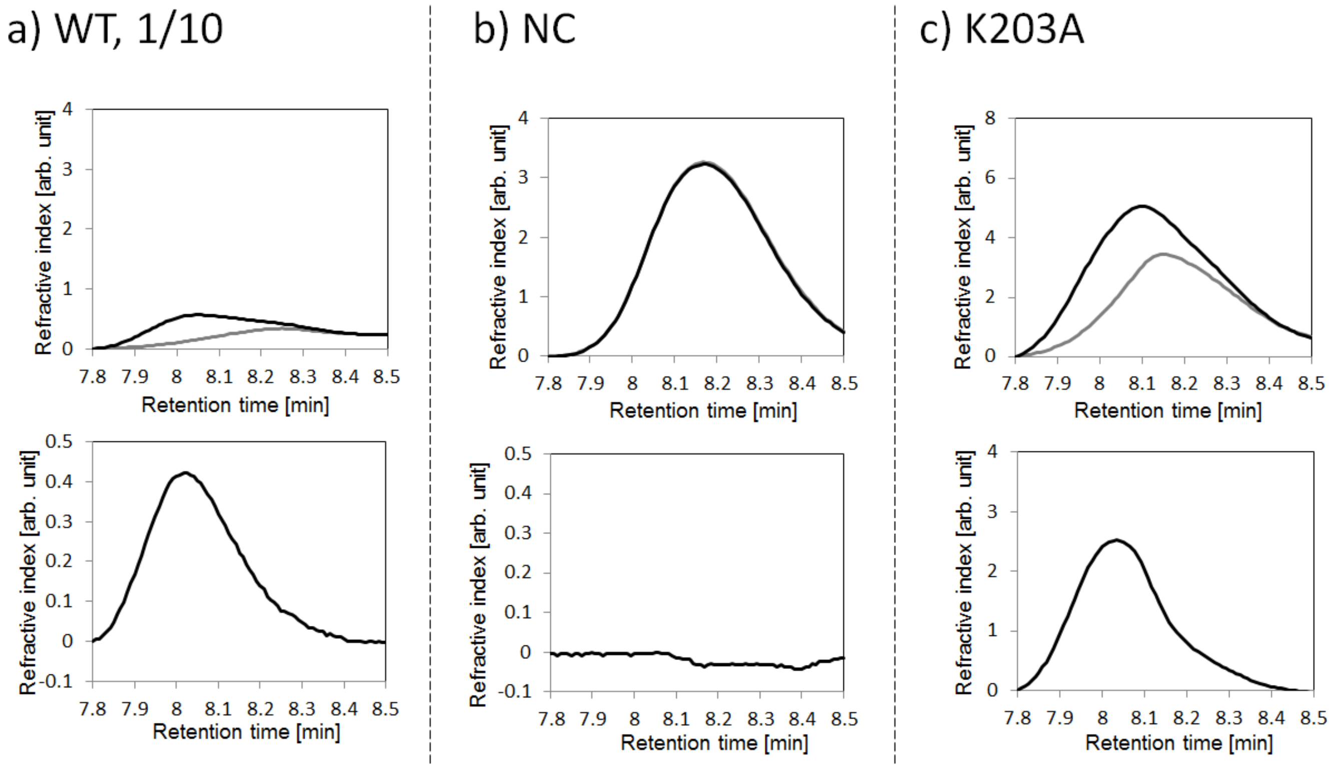

SUPPLEMENTARY FIGURE S10. Substrate threading in MD simulations of WT (Fig. 5b and d). (a) Time evolution of distance between the hydroxyl group oxygen atom and the N62 oxygen atom and (b) of the dihedral angle (N–C_{α}–C_{β}–C_{γ}) of N62.

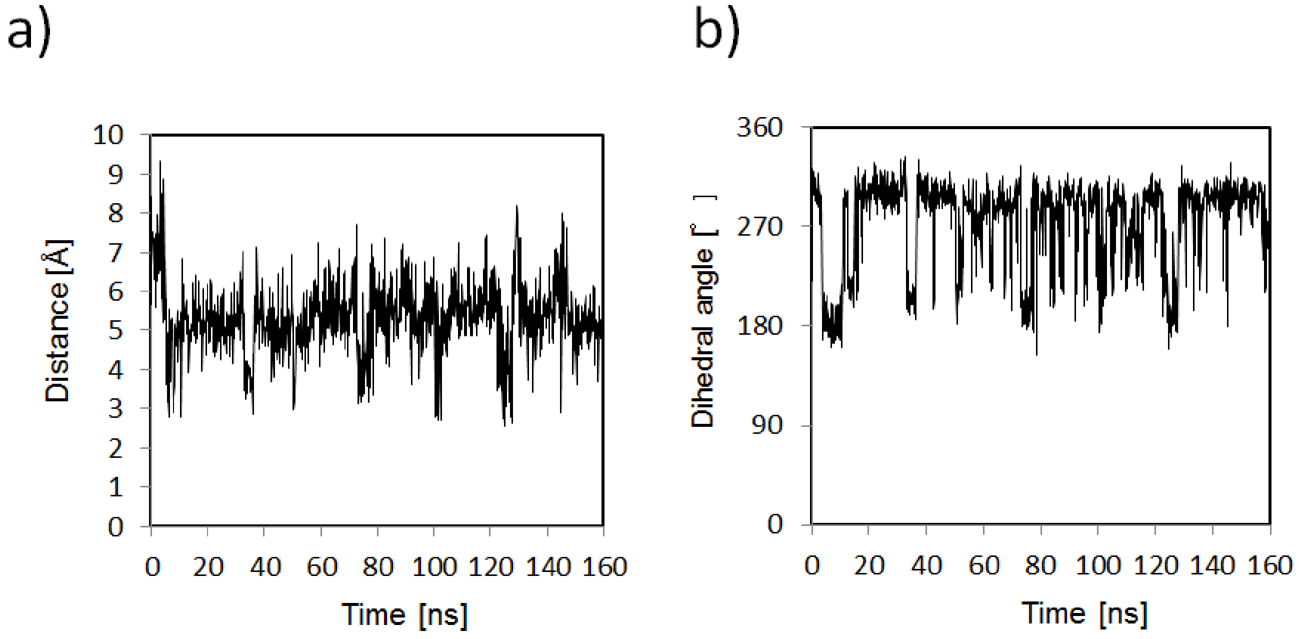

SUPPLEMENTARY FIGURE S11. MD simulations of the N222A mutant, using the same initial substrate setting as shown in Fig. 5a. Two time evolutions (black and grey lines) of the distance between the C1 atom of the reducing end glucose and C_α atom of catalytic base E234 in the MD simulation of WT are shown.

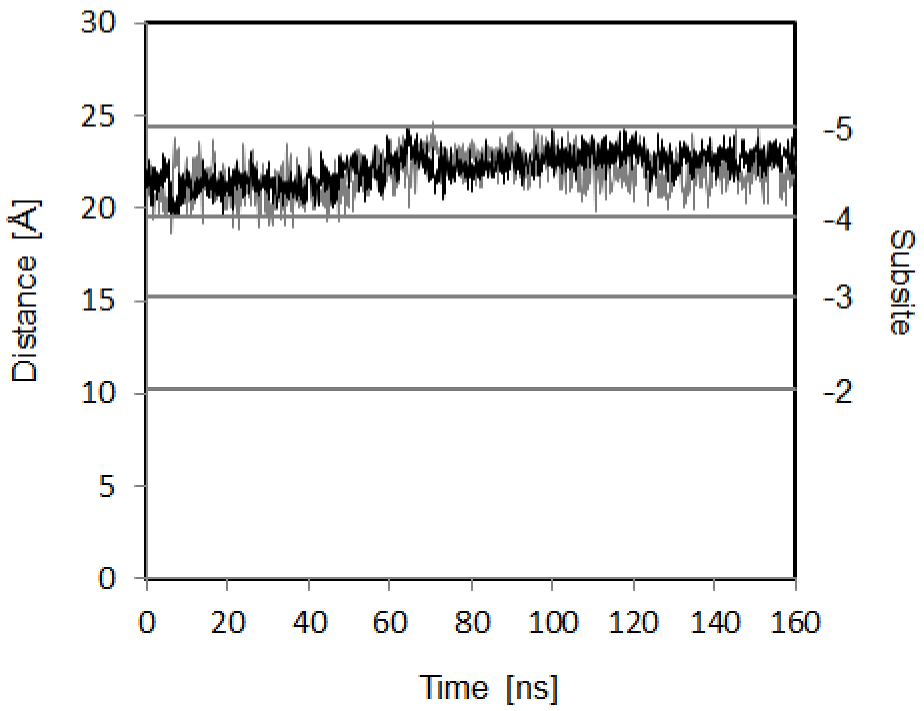

SUPPLEMENTARY MOVIE 1. Rotated homology model of *Tc*Cel7A (Fig. 1a).

SUPPLEMENTARY MOVIE 2. Substrate threading in MD simulations of WT, 1st run (Figs. 5b and d). The threading cellulose chain is represented by its constituent carbon/oxygen ring chain (thick orange stick). To indicate the position of each subsite, the cellulose strand from the homology model (Fig. 1a) is superimposed (thin yellow stick of carbon ring). N62 and W63 sidechains are shown as thick sticks (CPK colouring and purple, respectively).

SUPPLEMENTARY MOVIE 3. Substrate threading in MD simulations of the N62A mutant, 1st run (Fig. 5c and e) in the same manner as Supplementary Movie 2, but with A62 shown instead of N62.
